# Generation of a large compressive strain wave in graphite by ultrashort-pulse laser irradiation

**DOI:** 10.1063/1.5089291

**Published:** 2019-03-18

**Authors:** Xiaocui Wang, A. Jarnac, J. C. Ekström, Å. U. J. Bengtsson, F. Dorchies, H. Enquist, A. Jurgilaitis, M. N. Pedersen, C.-M. Tu, M. Wulff, J. Larsson

**Affiliations:** 1 Department of Physics, Lund University, P.O. Box 118, SE-221 00 Lund, Sweden; 2 MAX IV Laboratory, Lund University, P.O. Box 118, SE-221 00 Lund, Sweden; 3 Université de Bordeaux, CNRS, CEA, CELIA (Centre Lasers Intenses et Applications), UMR 5107, F-33400 Talence, France; 4 ESRF-The European Synchrotron, CS40220, 38043 Grenoble Cedex 9, France

## Abstract

We have studied strain wave generation in graphite induced by an intense ultrashort laser pulse. The study was performed in the intensity regime above the ablation threshold of graphite. The aim was to maximize the strain and, thus, also the internal pressure (stress). Laser pulses with a 1 ps temporal duration melt the surface of graphite resulting in a molten material which initially exists at the solid density. As the molten material expands, a compressive strain wave starts propagating into the crystal below the molten layer. The strain pulse was studied with time-resolved X-ray diffraction. At a temporal delay of 100 ps after laser excitation, we observed >10% compressive strain, which corresponds to a pressure of 7.2 GPa. This strain could be reproduced by hydrodynamic simulations, which also provided a temperature map as a function of time and depth.

## I. INTRODUCTION

Strain in graphite has been extensively studied in order to understand the role of non-thermal processes in strain generation^1–3^ and phonon-phonon interaction^4^ and to study heat transport in this anisotropic material.^5^ Graphite is one of the carbon allotropes. Their properties differ mainly due to the type of bonding, i.e., *sp*^2^-hybridized bonds in graphite and *sp*^3^-hybridized bonds in diamond.^6^ Short laser pulse irradiation has been used to synthesize novel carbon structures such as nano-diamonds^7,8^ and *sp*^3^-rich carbon nanofoams.^9^ However, the mechanisms of laser-driven phase transformation remain unclear on the ultrafast time scale.

The ablation threshold of graphite for 100 fs laser pulses at 800 nm has been reported to be 185 mJ/cm^2^.^10^ For fluences below this ablation threshold, Kanasaki *et al.* evidenced an *sp*^3^-bonded carbon nanostructure formed from graphite by multiple (10^4^) femtosecond laser pulses with a fluence of 64 mJ/cm^2^.^11^ They assigned the transformation process to coherent phonon motion involving interlayer compression, buckling, and shear displacement of graphite layers. Raman *et al.* used time-resolved electron diffraction (TRED) to observe interlayer compression in graphite induced by a femtosecond laser pulse with a fluence of 77 mJ/cm^2^, and their results suggest that an increased population of 2*p_z_* orbitals may induce interlayer attraction in the graphite lattice.^2^ Ultrafast laser ablation has been studied experimentally^10,12–14^ and theoretically^15^ at fluences higher than 185 mJ/cm^2^. Jeschke *et al.*^15^ found that ablation at lower fluence occurs without breaking the order within the graphene planes, but at higher fluences, these planes are also broken and new bonds are formed so that carbon clusters or chains emerge from the surface. In experimental studies, small quantities of nano-diamonds have been created following laser excitation in multi-pulse exposures.^7,16,17^ Micrometer sized patches of nano-diamonds covering less than 1% of the sample surface have been observed following a single-pulse exposure.^18^ A mechanism that may be involved in this phase transition is the compressive strain wave, which is launched by the ablation process and then propagates into the bulk graphite.

It is now possible to visualize structural dynamics in real time, thanks to the development of time-resolved X-ray diffraction (TRXD) and TRED techniques. So far, the application of these techniques to graphite has focused on strain measurements along the c-axis ([0 0 1]) at fluences below the ablation threshold.^1,3^ No direct time-resolved measurements have been carried out of the strain wave driven by ultrashort laser pulses in the intensity regime above the ablation threshold. Real-time studies of the laser-driven strain wave in the ablation regime would provide information that can contribute to our understanding of the dynamic transformation from graphite to diamond.

In the present experiment, we irradiated a single natural graphite crystal with an ultrashort laser pulse at a fluence above the regime in which Jeschke *et al.* found that the ablation products are chains or clusters of carbon atoms. We probed laser-induced strain in the lattice by TRXD. We observed interlayer compression of the lattice and deduced the internal pressure (stress) associated with the strain wave by analyzing the strain amplitude. We also evaluated the temperature of the graphite lattice using hydrodynamic simulations.

## II. EXPERIMENTAL SETUP

The experiment was carried out at beamline ID09 at the European Synchrotron Radiation Facility (ESRF). The sample was a single crystal of natural graphite, which was cleaved to a thickness of 20 *μ*m. The single crystal had an irregular shape with a size of approximately 2 mm × 2 mm. The small sample size and high in-plane thermal diffusivity of graphite ensure that the sample does not buckle due to a lateral non-uniform DC-heating. The sample was excited by pulses from a Ti:Al_2_O_3_ laser, with a central wavelength of 800 nm and a pulse duration of 1.2 ps. The maximum laser pulse energy was 2.6 mJ. The laser was aligned for normal incidence on the sample. The calculated reflectivity from tabulated values of the optical constants of graphite is 37% at normal incidence.^19^ The experiment was carried out in air. The laser spot on the sample was set to obtain the largest observable strain, and a damage size of 300 *μ*m × 300 *μ*m (H × V) was observed. At normal incidence, the ablation threshold of graphite is 185 mJ/cm^2^, which means that the absorbed fluence is >120 mJ/cm^2^. The reflectivity of graphite will increase after high-fluence laser irradiation^13^ due to the increased carrier density. This makes it difficult to obtain a value for the absorbed fluence, and we, therefore, considered the ablation threshold as a reference fluence. The area where the fluence exceeded the ablation threshold could easily be measured *in situ* using a sample microscope and was later verified *ex situ*.

The X-rays had a central energy of 15 keV with a 2.2% bandwidth and a pulse duration of 100 ps. The attenuation length of 15 keV X-rays in graphite is 6.6 mm,^20^ which means that most of the X-rays propagate through the thin sample. In order to avoid scattering from the sample mount, the graphite crystal was attached to a Kapton foil which was mounted free-standing on an aluminum frame. The incident angle of the X-rays was set to 12°, and the footprint size on the sample was 90 *μ*m × 290 *μ*m (H × V). The 2D X-ray diffraction pattern was recorded using a Rayonix MX-170 HS detector. The distance between the sample and the detector was 78 mm allowing for scattering measurements investigating the momentum transfer vector *q* up to 6 Å^−1^.

## III. EXPERIMENTAL METHOD

**FIG. 1.**
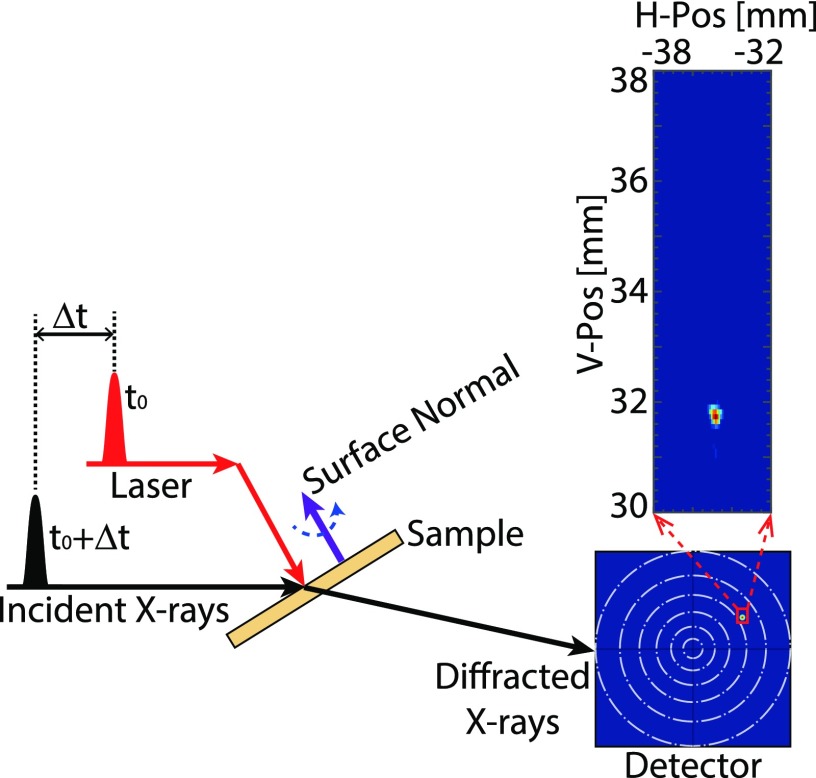
Sketch of the non-coplanar diffraction geometry. The surface normal of the graphite crystal is along the [0 0 1] direction. The dashed blue arrow shows the azimuth rotation. The dashed-dotted white circles on the detector indicate the values of the momentum transfer vector *q* (1–6 Å**^−^**^1^).

The experiment was performed in a non-coplanar diffraction geometry, as shown in Fig. 1. Graphite has a hexagonal lattice. In this paper, we use the abbreviated notation such as [0 0 1] for the c-axis. The *non-coplanar* (1 0 3) reflection was recorded. The angle between the (1 0 3) crystalline plane and the surface normal is 46.4°. The intensity of the (1 0 3) reflection was optimized by adjusting the azimuth angle. At an incident angle of 12°, the optimized azimuth angle for the (1 0 3) reflection is 79.8°, as shown in Fig. 1. We define the azimuth angle as the angle between the incident X-ray and the plane spanned by the surface normal and the [1 0 3] crystal axis. At this azimuth angle, the Bragg condition of the (1 0 3) reflection is fulfilled, as *θ_B_* = 15.6° for an X-ray energy of 15 keV. The large strain significantly changes the Bragg angle, and in order to study the strained material using a *coplanar* reflection, a scan over the incidence angle would be required. In the *non-coplanar* case, the Bragg condition could be fulfilled by an azimuthal scan of the sample. The width of an azimuthal scan was found to be 2° wide. The width is influenced both by the bandwidth and the mosaicity of the graphite crystal. If the material is strained by 15%, the peak azimuthal rotation is shifted by 1° in the geometry used in this study. That means that the highly strained material is still inside the FWHM range when the azimuthal rotation is kept constant at the peak value for the unstrained material. Hence, it was possible to use single-shot measurements since no parameters had to be scanned. However, in the actual experiment, the flux was too low to evaluate a single short. So each image is composed of several hundred laser shots which were acquired without scanning.

## IV. SIMULATION

**FIG. 2.**
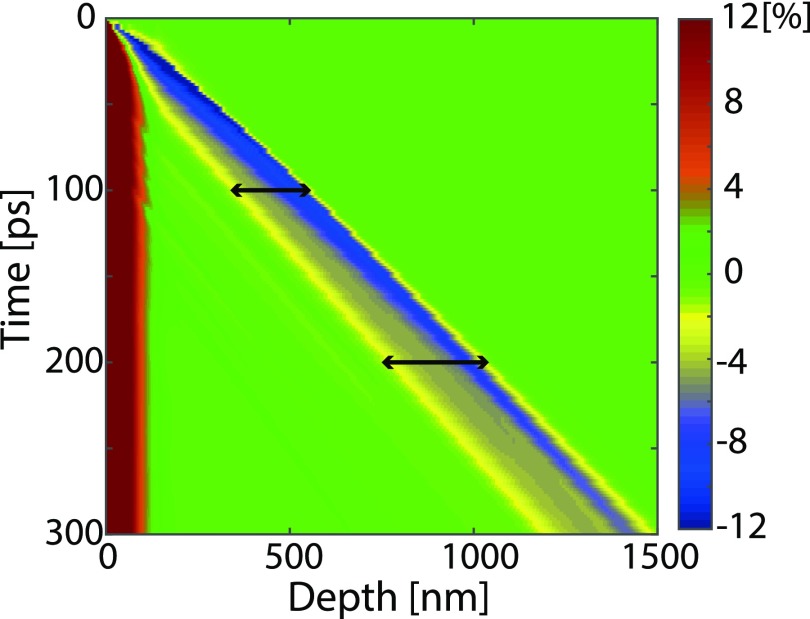
Simulated strain map as a function of time and depth. The black arrows indicate the width of the strain pulse with a compressive strain amplitude greater than 2% at both 100 ps and 200 ps.

When a solid target is illuminated by an intense femtosecond laser pulse, the target is heated to a hot fluid state, which dissipates by hydrodynamic flow.^21^ Shortly after laser excitation, the material is stressed since the broken bonds and elevated ionic temperature have created a material in a liquid phase at the solid density. This stress is released through propagating strain waves emanating at the liquid-vacuum and liquid-solid interfaces. Simulations of the generation and evolution of strain were performed using a 1D hydrodynamic code (ESTHER) which has been described by Leguay *et al.*^22^ and Colombier *et al.*^23^ The 1D hydrodynamic code solves the mass density evolution after laser illumination. The evolution of the mass density is governed by the diffusion of heat and fluid hydrodynamic equations coupled with a multi-phase equation of state.^23^ The underlying equations are given by Bushman *et al.*^24^ and by Lomonosov *et al.*^25^ In the simulations, the deposition of the laser energy was set to an exponential profile as a function of depth in the sample, with a width of 30 nm (1/e). In order to evaluate the strain from this output of the ESTHER code, the strain in graphite was defined as the relative change in the density: *S*(*t*) = *ρ*_0_/*ρ*(*t*) – 1, where *S* denotes the strain, *ρ*_0_ is the tabulated density of solid graphite (2.26 g/cm^3^), and *ρ*(*t*) is the density calculated by the hydrodynamic simulation. The strain profile at different times after the laser excitation was simulated in steps of 2 ps. Figure 2 shows the simulated strain map as a function of time and depth. By analyzing the data shown in Fig. 2, it can be seen that the strain wave initially propagates at a speed of 5000 m/s exceeding the longitudinal speed of sound along the c-axis which is 4140 m/s.^26^ The strain pulse is lengthened and the amplitude lowered during propagation. After 400 ps, the strain pulse is propagating at 4100 m/s. Strain-waves displaying supersonic velocities and a rapid drop in amplitude as the energy is used to heat the material are normally referred to as shockwaves.

**FIG. 3.**
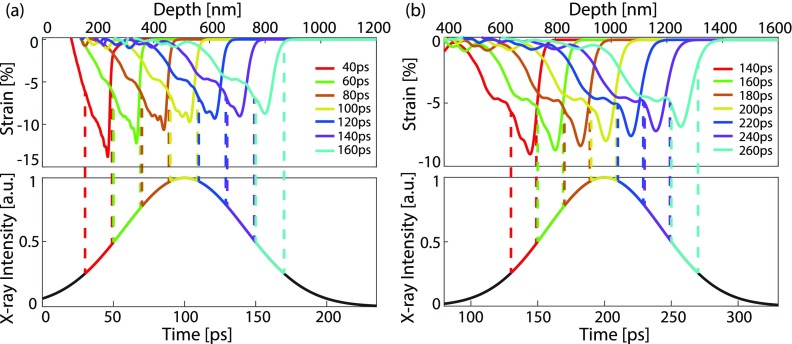
The strain pulse evolves during the 100 ps long X-ray pulse. To compare the simulated scattering to the measured signal, the strain pulse evolution is taken into account by calculating the scattered intensity at several times and adding them with a weight which equals the X-ray intensity at the corresponding time. The plots illustrate 7 separate time bins each represented by a color whereas the actual calculation was done in 45 steps. (a) The nominal time is 100 ps. (b) The nominal time is 200 ps.

The time-dependent calculated strain profiles provided the input for a non-coplanar X-ray diffraction code.^27^ This allowed for direct comparison to the experimental data. In order to directly compare the experimental and simulated data, we calculate the diffraction patterns taking into account the duration of the X-ray pulse. The duration of the X-ray pulse in the experiment was 100 ps. Lineouts from the ESTHER simulations are shown in Fig. 3. The strain evolves during the X-ray pulse, so we probe the different strain profiles shown in Fig. 3, with a weight corresponding to the X-ray amplitude at that time. The strain pulse shapes for two nominal time-settings (100 ps and 200 ps) are given in Fig. 3.

## V. RESULTS AND DISCUSSION

**FIG. 4.**
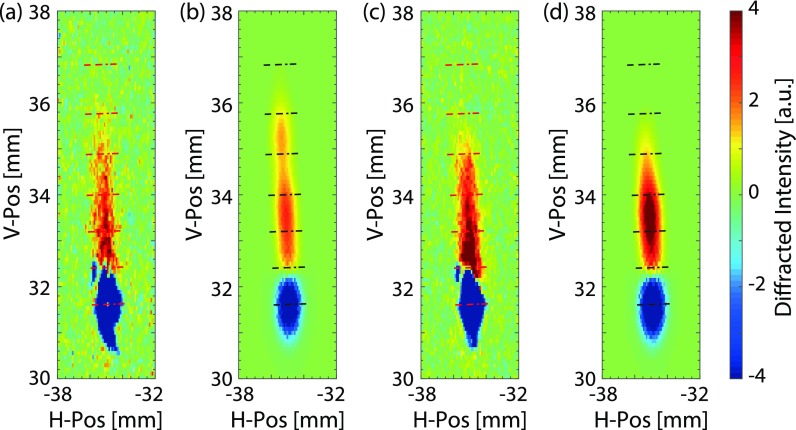
Difference in diffracted intensity from the graphite (1 0 3) crystalline plane after pulsed laser excitation: (a) experimental result at 100 ps, (b) simulation at 100 ps, (c) experimental result at 200 ps, and (d) simulation at 200 ps. The dashed lines represent a strain range of 0%–12% in steps of 2% along the c-axis of the lattice. Even if the peak strain at short times (40 ps) in Fig. 3(a) shows a strain exceeding 12%, the strain averaged over the X-ray pulse duration deduced from the data in Fig. 3 does not give a significant contribution above 10%.

X-ray diffraction patterns were acquired without laser excitation and at 100 ps and 200 ps after pulsed laser excitation. The diffraction images in Fig. 4 are difference images acquired with the pump laser on and off. The experimental difference images are shown in Figs. 4(a) and 4(c). At these time delays, the strain wave did not have time to reach the back surface of the sample. The results obtained from the simulations taking the X-ray pulse duration into account are shown in Figs. 4(b) and 4(d).

The negative values in Fig. 4 correspond to the position of the static (1 0 3) reflection. After laser excitation, the perfect lattice is perturbed, which leads to a decrease in the static reflection. The areas with positive values are the result of laser excitation. The laser-perturbed pattern shifted towards larger values of the momentum transfer vector in reciprocal space, indicating that the graphite lattice was compressed in real space after laser excitation.

Since the incident angle of the laser pulse is along the c-axis of the graphite lattice and because the lateral dimensions of the laser spot are much larger than the absorption depth, the strain wave compression is only along the c-axis during the short time scales considered here. The diffraction streak due to strain wave compression did not occur along a vertical line as seen in Fig. 4. This is due to the fact that the strain along the c-axis tilts the scattering planes.^28^

The magnitude of the strain can be derived from the scattering pattern by *S* = (*q* − *q′*)/*q*, where *q* is the value of the static momentum transfer vector. The maximum compressive strain of 10.0%±0.5% was observed 100 ps after laser excitation. The amplitude of the compressive strain decreased to 8.5% ± 0.5% at 200 ps. The uncertainty in the strain measurements arises from the energy resolution of the X-ray beam.

The pressure exerted by the strain wave can be extracted from the amplitude of the compressive strain wave via the bulk modulus. The bulk modulus of single crystalline graphite is 36.4 GPa according to measurements by Bosak *et al.*^26^ using inelastic X-ray scattering. However, the compressibility of graphite is non-linear, and we use the study by Lynch and Drickamer^29^ to deduce the pressure corresponding to the measured strain. We determined the pressure exerted by the strain wave to be 7.2 GPa 100 ps after laser excitation. With a 200 ps delay between the pump and probe, the pressure was reduced to 4.8 GPa.

The diffraction patterns in Fig. 4 provide information that can be used not only to evaluate the strain but also to estimate the number of strained layers for a given range of strains. This is due to the fact that the intensity increases with the number of layers with a particular interlayer spacing. We assumed that the graphite crystal is an imperfect crystal and that the diffracted intensity is proportional to the thickness.^30–32^ We used the 20 *μ*m thickness and the intensity of the diffraction spot without laser excitation as a reference. At 100 ps, the thickness of layers strained more than 2% is 210 nm and increases to 260 nm at 200 ps. These layers are represented by the black arrows in Fig. 2. Since the diffraction intensity is proportional to the number of strained layers, the thickness of the strained material was evaluated from the experimental data, giving values of 360 ± 20 nm at 100 ps and 500 ± 25 nm at 200 ps, which are in reasonable agreement with the simulations. From the X-ray diffraction pattern in Fig. 4, it is possible to follow the early development of the strain wave as it propagates through the material. It can be seen that as the length (maximum strain) of the X-ray spot is reduced, the diffracted intensity at intermediate strain (∼5%) is higher at 200 ps compared to that at 100 ps. This is because as the strain relaxes, more layers with lower values of strain contribute to the signal, which means that the width of the strain wave increases. The increase in the width of the strain wave is also evident from the simulations shown in Fig. 2.

**FIG. 5.**
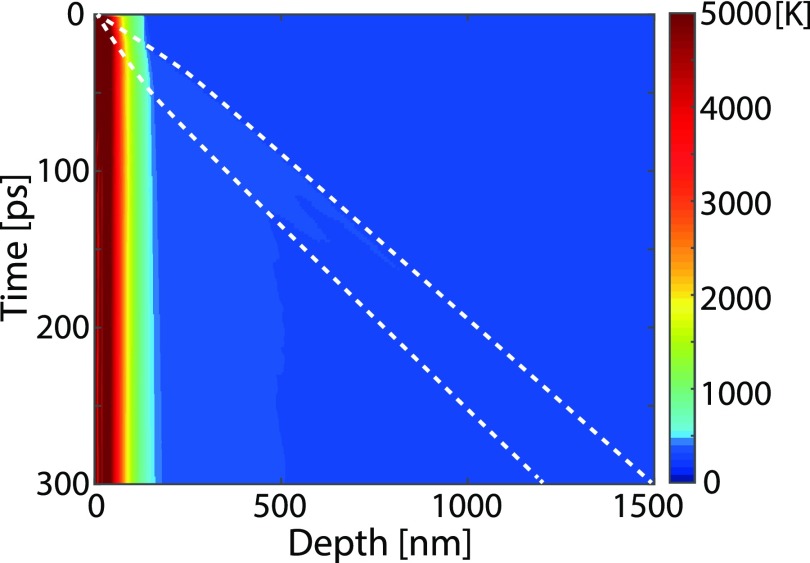
Simulated temperature map as a function of time and depth. The white dashed lines delineate the strain wave region where the compressive strain amplitude is greater than 2%.

We have extracted the pressure induced by intense ultrashort laser pulses. To interpret the experimental conditions in the context of the carbon phase diagram, information is required on the temperature, which was provided by the hydrodynamic simulations, and shown in Fig. 5. The inserted white dashed lines delineate the strain wave region where the compressive strain amplitude is greater than 2%. As seen in Fig. 2, the strain wave has travelled about 400 nm within the first 100 ps after laser excitation. The simulation results shown in Fig. 5 yield that the temperature of the lattice is still below 315 K. The boundary between graphite and diamond at room temperature in the carbon phase diagram has been reported to be at a pressure of 2.3 GPa (Bundy *et al.*^33^). In the present experiment, we found the pressure to be 7.2 GPa, 100 ps after pulsed laser excitation. The pressure and temperature are, thus, in the diamond region of the carbon phase diagram, indicating that the phase transformation from graphite to diamond could occur under these conditions.

Using time-resolved electron diffraction measurements, Raman *et al.*^2^ observed a 6% transient change in interlayer compression 14 ps after laser excitation and that the transient structure recovered to *sp*^2^ character by 45 ps. In the present experiment, with a higher laser excitation fluence, we observed 10.0% interlayer compression, i.e., nearly twice that observed by Raman *et al.* Our experiment setup is limited by time resolution, and it was not possible to access the interlayer compression at earlier times. Nonetheless, the 10.0% interlayer compression observed in this study persisted twice as long (100 ps) as that reported by Raman *et al.* To achieve a 10% interlayer compression, a 7.2 GPa pressure is required. Since the bulk modulus is 36.4 GPa, we have reached a regime with a non-linear stress-strain relationship. In the ESTHER simulations, we can see characteristics of a shockwave, namely, a broadening of the pulse and a supersonic propagation velocity. The pulse broadening is observed in the experiment, but the temporal resolution at ESRF (100 ps) and the rapid dissipation to a sonic wave which according to the simulations occur in 400 ps do not allow us to experimentally verify supersonic propagation velocities.

## VI. CONCLUSION

In conclusion, we have observed >10.0% compressive shockwave in a graphite crystal, corresponding to a pressure of 7.2 GPa, with a time resolution of 100 ps. By performing hydrodynamic simulations, we estimated that the temperature was below 315 K. The combination of pressure and temperature is in the diamond region of the carbon phase diagram. This means that the strain/pressure wave may drive the phase transformation from graphite to diamond, which was observed in the work by Nüske *et al.*^18^ Nüske *et al.* discussed three potential mechanisms for diamond formation. The first is a stress driven phase transition where high pressure could yield a pathway to diamond. The second one is a more atomistic mechanism involving coherent phonon motion which is supported by experimental findings by Kanasaki *et al.*^11^ and theoretical predictions by Garcia *et al.*^34^ based on tight binding molecular dynamics (TBMD) calculations. The mechanism in this case is a restacking to AAA graphite followed by buckling of planes and formation of *sp*^3^ bonds. A third suggested mechanism is that diamonds are formed during rapid quenching of liquid carbon.^35^ Further studies should be carried out on time-scales shorter than 100 ps in order to identify the mechanism and gain information on the early stages of the pressure change in graphite.
